# The effect of EEG neurofeedback on lowering the stress reaction level depending on various stressors on the biochemical, muscular and psychomotor sphere: A preliminary randomized study

**DOI:** 10.1097/MD.0000000000037042

**Published:** 2023-02-02

**Authors:** Marcin Dornowski, Dominika Wilczyńska, Milena Lachowicz, Inna Sokolowska, Tomasz Szot, Robert Urbański, Andriy Maznychenko, Andrzej Szwarc, Kacper Gotner, Dominik Duda, Piotr Sawicki, Jakub Hinca

**Affiliations:** a Gdansk University of Physical Education and Sport, Gdansk, Poland.

**Keywords:** brain waves, human, muscle, psychology, stressor

## Abstract

**Background:**

The human body is exposed to stressors on a daily basis. Short-term exposure to a particular stressor can cause the release of inflammatory markers – including c-reactive protein (CRP). EEG neurofeedback is a noninvasive form of therapy that aims to improve brain function. Neurofeedback is a type of feedback based on brain activity.

**Methods:**

The research process was performed by a group of 80 men who were divided into 2 research groups and 2 control groups. In the first research group and the first control group, the stressor of high temperature was applied, while in the second research group and the second control group, the stressor was physical exertion to refusal. Meanwhile, blood samples were taken to visualize inflammatory markers. These were taken before and after the stressor, as well as before and after the application of EEG neurofeedback.

**Results:**

In research group after EEG neurofeedback intervention, the level of CRP significantly dropped in the measurement after stressor implementation. Analysis of the *SMK* test revealed a significant influence of both factors (time F = 13.525, *P* = .035; state F = 10.658, *P* = .047) and their interaction (F = 16.709, *P* = .026). Same statically significant decrease was observed in the level of rect. abdom. EMG was after physical work after neurofeedback. In all tests, a decrease in the EMG amplitude of *upper trap.* was observed after physical work before neurofeedback with its further increase after neurofeedback. After neurofeedback training, the results of the 3 tested parameters of the sensorimotor coordination test performed after the second heat stressor improved statistically insignificantly compared to the values obtained before.

**Conclusion:**

This article shows the effect of EGG neurofeedback on reducing the negative effects of stress exposure in humans. The study showed that the level and a pattern of EEG neurofeedback influence and significance is different depending on the applied stressor. Furthermore, the level of EEG neurofeedback influence and significance in decreasing the stressor effect is different depending on the examined sphere.

## 1. Introduction

Throughout daily physical responses, muscles are exposed to stressors, resulting in a demand for energy and contractile activity. Every physical activity indicates stressors, but not only. There is a tendency for multiple cases in the human body where the stressors might affect the body function and life.^[1]^ One of these stressors is a change in temperature that can increase with work intensity and the metabolic rate. An answer to stressors in an organism can be twofold. For instance, exposure to the heat stress frequently increases both the protective heat shock proteins (HSPs), and the transcriptional co-activator, peroxisome proliferator-activated receptor gamma, coactivator-1 alpha (PGC-1a) which is crucial in terms of adaptation.^[2]^ However, Mikicin et al^[3]^ have shown that stressor usage appears to be more beneficial due to the reduction of the negative stress exposure, which can be achieved through neurofeedback training. In electroencephalography biofeedback therapy (EEG) for swimmers, the evidence has shown significant improvements in the working rates in comparison to the control group. Olympic athletes who were preparing for Vancouver 2010 Olympics managed to perform better due to EEG and psycho-physiological stress assessment as well as a bio–neurofeedback training intervention.^[4]^ Also, in soccer players who have been training twice a week for 4 weeks with EEG, it was proven that the neurofeedback group finished with fewer injuries.^[5]^ Therefore, the muscular effects of bio-feedback are inarguable and already used in professional sports. Nonetheless, sport is not the only area of human activity that can implement new ways to take care of productivity and health. Physiological muscle activity tends to be higher in the individuals who are active, which determines a faster response to stressors. Therefore, trained subjects have a raised base concentration of creatine kinase, alanine transaminase, and lactic acid dehydrogenase, and this may reflect an adaptation to regular training.^[6]^ This is similar to the brain, which can also adapt to stressors.^[7]^ The central nervous system adaptation to stressors is vital because of the early influence of stress in human development. It has been proven that stressful events in early life frequently affect a person behavior or development. In recent years posttraumatic stress disorder has gained more attention and shows that there are possibilities of a more effective counteraction to the disturbance. For instance, there are significant effects of neurofeedback treatment on posttraumatic stress disorder.^[8]^ That indicates a beneficial field in terms of the advantages of using neurofeedback actions for the mental realm. Although there is a lack of research in the field of neurofeedback and genetic adaptation, it could be beneficial to explore this problem further. The studies conducted among spinning instructors have shown genetic adaptation to the training; however, authors concluded that the spinning instructors might feel overtrained due to a lack of post-workout regeneration. Moreover, it is believed that research in the field of EEG bio-feedback can also be beneficial. Biofeedback is a training tool that measures physiological changes associated with how a person is feeling, thinking and behaving. By feeding this information back in real time, biofeedback creates a context for learning to regulate one own body processes and thereby influence one emotional resilience, etc. Neurofeedback is a special form of biofeedback, a form of brain training that works directly at the physiological level. It aims at improving brain performance and thereby such functions as: the executive function: attention, focus and concentration, also impulse control and self-organization; emotional self-regulation or emotional resilience; motivation, willpower and energy. Neurofeedback works specifically with measures of brain activity (as opposed to more “peripheral” measures, such as muscle tension or the heart rate variability).^[9–11]^ In view of the above, the aim of the study was to define the level of influence of neurofeedback sessions on stress model profiles experienced during physical effort and high temperature stressors according to biochemical, muscular, and psychomotor signatures. The following hypothesis was put forward in the paper: EEG neurofeedback sessions will significantly lower the level of stress experienced from maximal physical effort and high temperature as also reflected in the biochemical, muscular, and psychomotor profiling areas. The level and the pattern of this influence will be different according to applied stressor.

## 2. Materials and methods

### 2.1. Participants

The participants were 80 healthy males students from Gdansk University of Physical Education and Sport divided into 4 groups. In the first experimental and control group, a stressor (high temperature) was used, in the second experimental and control group – fatigue exercise to failure. The characteristics of the groups are presented in Table 1. Each of the subjects expressed their consent to participate in the research in writing. The conducted research also has the consent of the Bioethics Committee (KB – 3/21).

**Table 1:** Characteristics of the participants.

Characteristics	Group 1 experimental	Group 1 control	Group 2 experimental	Group 2 control
No. of subjects	20	20	20	20
Age (yr)	22.2 ± 0.95	21.3 ± 0.54	25 ± 4.69	23 ± 4.12
Height (cm)	185.5 ± 4.65	180.5 ± 3.13	178.7 ± 4.57	179.6 ± 3.15
Bodyweight (kg)	92.8 ± 12.79	90.7 ± 10.64	86.6 ± 6.89	85.4 ± 3.25
BMI	27.05 ± 1.22	26.03 ± 0.22	27.2 ± 2.76	25.1 ± 1.65
Skeletal muscle mass (kg)	42.4 ± 10.84	40.1 ± 9.15	39.4 ± 7.03	38.3 ± 6.07
Body fat mass (kg)	18.7 ± 3.96	16.6 ± 2.42	17.5 ± 2.89	16.2 ± 3.15

### 2.2. Methods

Biochemical evaluation (inflammation marker CRP connected with stress reaction of human cells): blood samples were collected before and after stressors as well as before and after EEG neurofeedback implementation.^[12,13]^ The aim of the research task was to assess the inflammatory state, which is strongly related to the broadly understood perception of stress in the psycho-physical sphere. CRP is used as a clinical marker of inflammation which is also strongly associated with cardiovascular functions. Both stressors used in the research were connected with this system.^[14–17]^

#### 2.2.1. Muscular evaluation.

The EMG signal was recorded from selected muscle groups during VTS (Vienna Test System) measurements – *m. rectus abdominis (rect. abdom.), m. upper trapezius (upper trap.).* It allows precisely registering the duration of latent voluntary contraction and relaxation and can be used for assessment of the intensity of central commands that control muscle contraction. The EMGs were collected via TeleMyo DTS 2400 System (16 channels) with MyoResearch Master Edition software. The electrodes were placed on the selected muscles: m. Rectus abdominis (rect abdom), m. Upper trapezius (upper trap). Measurements and procedures were carried out according to SENIAM principles.^[18]^

#### 2.2.2. Psychomotor evaluation.

A battery of the following Vienna Test System tests was used. CORSI measures the storage capacity of spatial working memory. The Block-Tapping Test is regarded as the golden standard for testing spatial memory span. SMK (sensorimotor coordination test) assesses eye-hand, hand-hand and eye-hand-foot coordination in 3-dimensional space, where movements are controlled by utilizing the feedback of sensorimotor information from the movement currently being executed. SIGNAL measures long-term focused attention and visual differentiation of a relevant signal when distractor signals are present; the signal detection theory describes the perception of weak signals against a constantly changing background. DT (determination test) assesses reactive stress tolerance and ability to react under complex stimulus conditions. DT is a complex multi-stimuli reaction test involving presentation of both colored stimuli and acoustic signals to which the respondent reacts by pressing appropriate buttons on the response panel and using foot pedals.

#### 2.2.3. EEG neurofeedback intervention.

The session protocol involved 4 tasks: baseline eyes open, baseline eyes closed, sensory attentiveness, cognitive effort. During the protocol, influence was exerted on the alpha, beta, theta and specialized mobile radio waves and on the values of theta/alpha, theta/beta and specialized mobile radio/theta waves ratios. Neurofeedback sessions (relaxation treatment protocol) were done via ProComp5 Infiniti Biomed. There were 2 connections with ear lobe clips and one connection on the subject head, in the middle of the ear line. All sites were prepared with exfoliating paste prior to each session, and the conductive paste was used for the head electrode. EEG neurofeedback sessions were implemented in both groups (in the one where the stressor was maximum effort to refusal and high temperature). Each of the participants performed 10 individual sessions. The first session was performed on the day following the first phase of laboratory measurements. After the last day of the last EEG neurofeedback session, a second round of laboratory measurements was performed.

### 2.3. Stressors

*Stressor 1: high temperature* – sauna was administered 2 × 15 minutes at a temperature of 100°C with a 10-min break.

*Stressor 2: fatigue exercise to failure* – A cardiopulmonary exercise test was carried out using a mechanical treadmill from the h/p cosmos Saturn company (Table 2).

**Table 2:** Procedure protocol.

Interval	Time [min]	Velocity [km/h]	Angle [%]
Rest	3	0	0
1	3	2.7	10
2	3	4.0	12
3	3	5.4	14
4	3	6.7	16
5	3	8.0	18
6	3	8.8	20
7	3	9.6	22
Restitution	3	0	0

### 2.4. Study design and procedure

**Figure 1.**
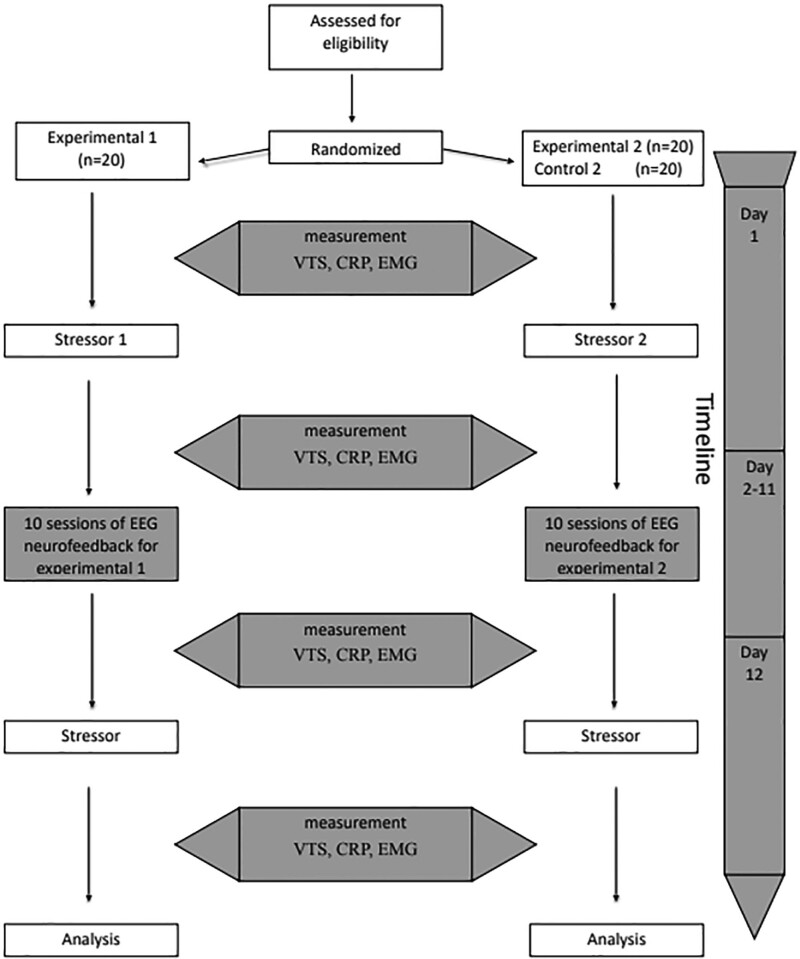
Study design and procedure.

The experiment was performed on 2 groups of students from Gdansk University of Physical Education and Sport. The students were recruited through the University website. After the meeting, 20 students were randomly assigned to group 1 experimental and 20 to group 1 control group (high temperature intervention) and 20 students to group 2 experimental and 20 to group 2 control group (fatigue exercise to failure). All the participants signed a formal agreement as well as officially confirmed their lack of health problems to participate in the experiment. The purpose of the experiment was to apply 10 sessions of EEG neurofeedback to participants who were exposed to 2 different stressors (extreme conditions): high temperature (sauna) and fatigue exercise to failure and assigned to experimental groups. In the initial phase of the experiment, all subjects were subjected twice to 4 selected test from the Vienna Test System (CORSI, SMK, SIGNAL, DT) together with EMG evaluation, biochemical evaluation – blood samples: the expression of genes HSF1, HSPA1A, HSPB1, NF-κB, CCL2, IL-10, IL-6, HIF1A, and CRP, and psychological tests. Then they were subjected to stressors: group one – high temperature (2 sauna visits for 15 minutes with a 10 to 15 minutes break, temperature 100°C); the second group performed fatigue test/exercise to failure on a cyclo-ergometer according to the Bruce protocol, followed by a second application of selected tests. In the second phase, each participant was subjected to 10 sessions of EEG neurofeedback, and then the selected tests measurements were re-performed before and after the application of the stressor (Fig. 1).

Statistical analysis showed that in the group of many analyzed parameters, several showed changes between the levels before and after the experimental intervention (EEG neurofeedback session) with statistical significance. Therefore, in the further part of this work, only those results that was confirmed as statistically significant will be presented. These studies are pilot studies, the results of which are to be used as a way to select key parameters for the next stages of using the procedure of this project. The statistical analysis was performed reliably and with all rigor.

## 3. Results

### 3.1. Biochemical sphere

**Figure 2.**
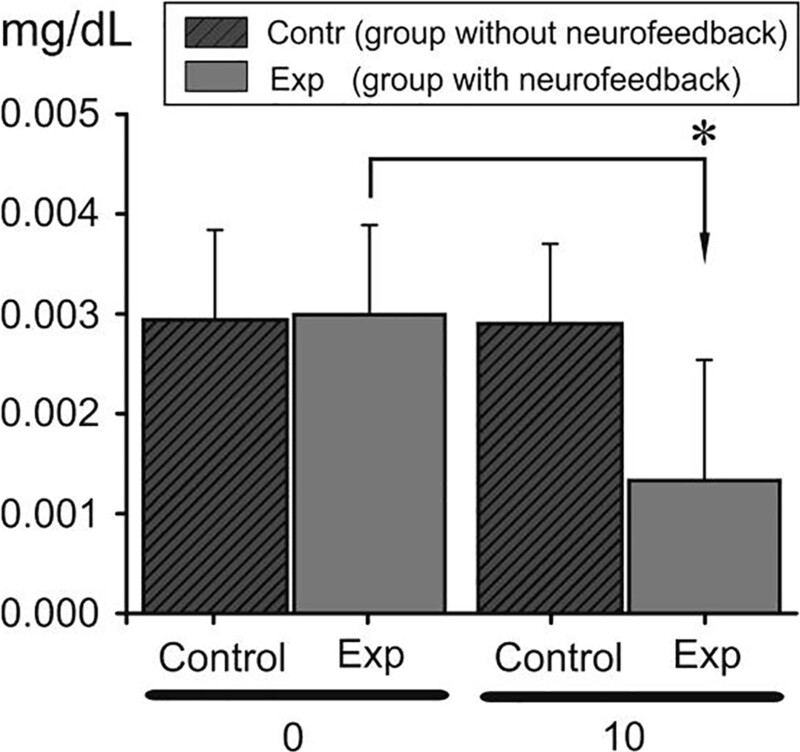
CRP expression in high temperature stressor group (group 1 experimental and control) before (0 d, dark gray column) and after (10 d, gray column) EEG neurofeedback sessions. The asterisk denotes significant differences (*P* < .05). CRP = c-reactive protein.

Gene expression was investigated in PBMCs of 80 healthy men who underwent different stress conditions: high temperature (20 experimental and 20 control) and maximal physical effort (20 experimental and 20 control). It was observed that the expression of the CRP gene was significantly higher after the heat stress as compared to the baseline level during non-stressor conditions. However, after EEG neurofeedback intervention, the level of CRP significantly dropped in the measurement after heat stressor implementation. Results achieved by control group in both stressors did not show significant changes (Fig. 2).

### 3.2. Muscular sphere

To estimate possible dependences of the EMG amplitude on the experimental condition in both groups (with physical effort or high temperature), we applied 2-way statistical analysis of variance with repeated measurement. The factors of variation included 2 conditions: time (two levels: 0 – before neurofeedback and 10 days – after neurofeedback) and state (two levels: before and after physical workout or high temperature). Values with *P *< .05 were considered to be significant.

**Figure 3.**
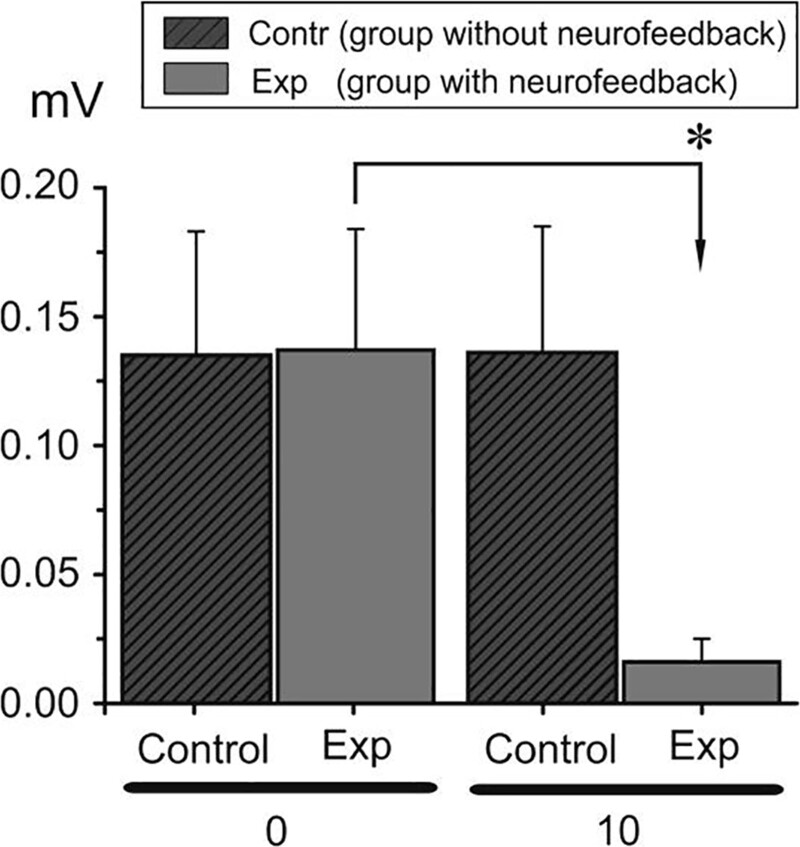
Averaged characteristics of the EMG activity of *rect. abdom.* in physical effort stressor group 2 experimental and control before (0 d – dark gray column) and after neurofeedback (10 d – gray column). The asterisk denotes significant differences (*P* < .05). EMG = electromyography.

A Bonferroni post hoc analysis was used when a significant difference was detected. In group 1 experimental, the investigated muscles did not exhibit dependence on any of these factors, except activity of *rect. abdom.* during realization of the *SMK* test (Fig. 3). In this case, analysis revealed a significant influence of both factors (time F = 13.525, *P* = .035; state F = 10.658, *P* = .047) and their interaction (F = 16.709, *P* = .026). A statistically significant increase in amplitude of *rect. abdom.* was observed after exercise, both before and after neurofeedback. In contrast, the level of EMG activity of this muscle was significantly lower before physical work after neurofeedback compared with that before neurofeedback. The same statically significant decrease in the level of *rect. abdom.* EMG was observed after physical work after neurofeedback compared with that before neurofeedback (group 1 experimental, *SMK*). The data obtained in group 1 experimental shows a similar dynamic in EMG amplitude changes of *rect. abdom.* during realization of the *CORSI, SIGNAL, DT* tests as in the *SMK* one. It should be noted that in these tests the level of *rect. abdom.* EMG activity was higher before and after physical activity after neurofeedback, but a significant dependence on any of the investigated factors was not revealed. In all tests, a decrease in the EMG amplitude of *upper trap.* was observed after physical work before neurofeedback with its further increase after neurofeedback. In group 2 experimental, during the *DT* tests, *rect. abdom.* activity was more expressed, while the *upper trap.* muscle was active in the *CORSI, SIGNAL* and *SMK* tests. Statistical analysis did not show a significant influence of temperature and neurofeedback on the EMG activity of the investigated muscles. Results achieved by control group in both stressors did not show significant changes.

### 3.3. Psychomotor sphere

**Figure 4.**
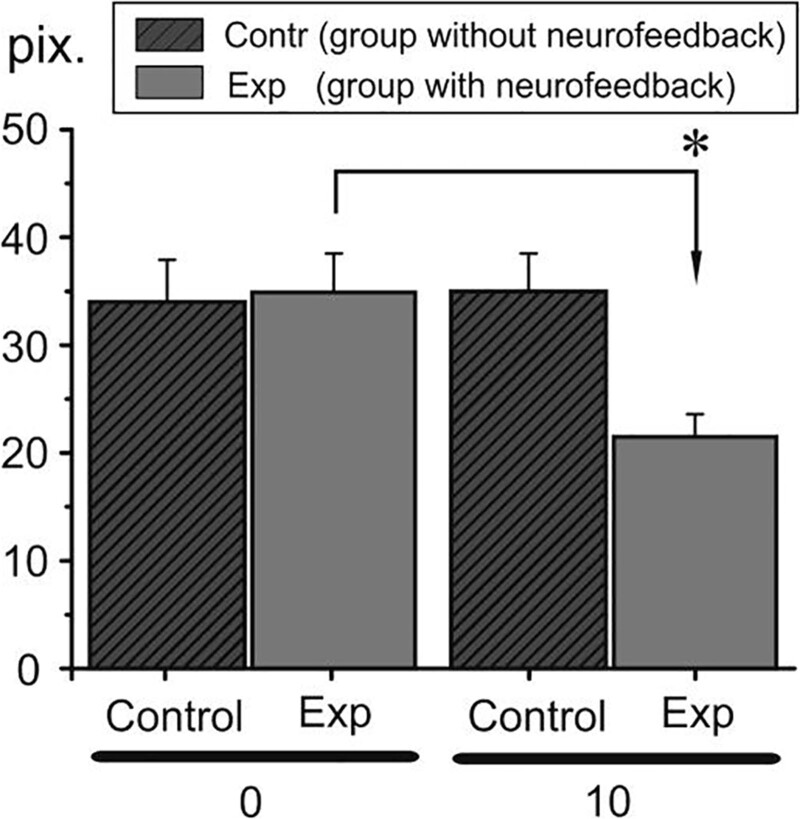
The SMK test mean values of the horizontal deviation in the high temperature stressor group (group 1 experimental and control) before (0 d – dark gray column) and after (10 d – gray column) EEG neurofeedback sessions. The asterisk denotes significant differences (*P* < .05). SMK = sensorimotor coordination test.

In the group of subjects exposed to the first stressor in the form of a sauna visit (group 1 experimental), the values of the 3 examined parameters of the SMK test slightly improved in comparison to the results of the coordination test before the application of the high temperature stressor, but the difference was not statistically significant. After the neurofeedback training, the results of the SMK test carried out before the second stressor improved compared to the SMK test carried out before the neurofeedback training before the first stressor. Concerning the mean value of the angular deviation, the difference was statistically insignificant, while in the case of the mean value of the horizontal deviation, these differences were statistically significant (t = 3.50; t = 4.160). Comparison of the results of the SMK test carried out after the application of the first stressor with the results of the SMK test carried out after the neurofeedback training and after the second stressor showed an improvement in the coordination parameters. In the case of the mean value of the angular deviation, it was statistically insignificant, while the recorded difference between the mean value of the horizontal and vertical deviation was statistically significant (t = 4.07; t = 2.56). After neurofeedback training, the results of the 3 tested parameters of the sensorimotor coordination test performed after the second sauna visit improved statistically insignificantly compared to the values obtained before using the sauna for the second time (Fig. 4). Results achieved by control group in both stressors did not show significant changes.

## 4. Discussion

During EEG neurofeedback experiment intervention, almost all subjects have a decreased factor of the alpha wave responsible for rest, relaxation and creativity, which may be related to the lack of calmness and rest. Noteworthy is a very low alpha wave index in the case of one subject (is this a symptom of exhaustion?). With low alpha a person may have low self-esteem and weaker motivation. This is a preliminary study, which investigated acute effects on biomarkers of stress and inflammation of heat stress (sauna) and incremental exercise (maximal aerobic testing) in healthy men. CRP is an acute phase protein released mainly by hepatocytes, but also by smooth muscle cells, endothelial cells, lymphocytes, macrophages, and adipocytes. The CRP levels are known to increase dramatically in response to injury, tissue damage, infection, and inflammation. It was already observed that CRP concentration increases 4 to 6 hours after acute tissue injury or inflammation and decreases rapidly with the removal of the causative agent.^[1]^ Our study also confirms the above investigation and reveals an increase in the CRP mRNA level already 3 hours after the sauna. Moreover, CRP concentration continued to increase up to 6 hours after the stressor. The literature on the subject shows research on determining the level of dependence of general inflammation determined by the level of CRP and the level of the stress response of the respondents. The dependence of these variables was shown. Chronic stress increased the value of CRP.^[19,20]^ Earlier, it has been shown that biofeedback is effective for symptom reduction in some clinical conditions,^[21]^ for example, pain,^[22]^ epileptic seizures,^[23]^ and attention deficit-hyperactivity disorder,^[24]^ as well as for improving motor performance after stroke.^[25]^ Bazanova et al^[26]^ also showed a positive efficiency of neurofeedback with EMG training in ADHD children. Enhanced muscle tone is thought to be a sign of psycho-emotional tension or mental stress.^[18–26]^ In our study, a decrease in *rect. abdom.* EMG activity can indicate some relaxation after neurofeedback and thus less muscle tension in response to stress. In this case, we can also speak about a positive effect of neurofeedback on muscle activity under the influence of stress factors. In the studies taking into account the use of EMG analysis, the authors indicate an increase in the total electrical activity of the analyzed muscles in a stressful situation. One of the aims of the current study was to analyze the influence of 2 independent stressors on selected cognitive parameters of respondents and if those parameters changed after the implementation of neurofeedback training in 2 conditions (sauna stressor and physical workout stressor). The results showed some significant changes in the SMK form of VTS, while there were no significant changes in other cognitive parameters (working memory, stress tolerance, attention) after the neurofeedback training sessions. In the group of respondents exposed to the stressor of physical workout, the mean value of the horizontal deviation parameter of SMK improved after the neurofeedback training comparing to the group of participants exposed to the sauna conditions. However, there was a statistically significant improvement in the mean value of the horizontal and vertical deviation after neurofeedback implementation in the group with sauna conditions. The use of the tests of the Vienna Test System to determine the degree of stressor impact and, consequently, the potential possibilities of implementing difficult coordination tasks have been shown, among others, in studies of Kiss et al^[27]^ An interesting study on various types of neurofeedback is the work Marzbani et al^[28]^ in which we read about various ways of using it and a scientific approach. The authors objectively indicate the advantages and disadvantages of the method, however, they “urge” for further scientific exploration. Despite the lack of studies describing the effect of neurofeedback training on the results of the sensorimotor coordination, there have been articles analyzing the use of this intervention in order to improve coordination skills. For example, in one study,^[29]^ the authors analyzed the possibility of using the neurofeedback method to optimize the timing and technique of microsurgical procedures among surgeons, but the results were inconclusive On the other hand, Norouzi et al^[30]^ analyzed the effectiveness of neurofeedback training in improving the coordination abilities of children with ADHD and observed a significant improvement in motor control parameters and fewer errors in the 2-handed wrist coordination test. In a meta-analysis, Xiang et al^[31]^ pointed out that the implementation of neurofeedback training among athletes significantly improved sports results.

## 5. Conclusions

The level and a pattern of EEG neurofeedback influence and significance is different depending on the applied stressor. The level of EEG neurofeedback influence and significance in decreasing the stressor effect is different depending on the examined sphere. The achieved results allow incorporating EEG neurofeedback sessions in specific professional activity and stressors which could appear there. In the biochemical sphere, the CRP values before and after the experiment, allow using EEG neurofeedback sessions in maintaining physical efficiency when a high temperature stressor is implemented. In the psychomotor sphere, the SMK test values before and after the experiment allow using EEG neurofeedback sessions in maintaining movement coordination when a high temperature stressor is implemented. In the muscular sphere, EMG (*rect. abdom.* electrical activity values) before and after the experiment allow using EEG neurofeedback sessions in maintaining movement reactivity when a maximal physical effort stressor is implemented.

## Acknowledgments

During the research, there was no external support.

## Author contributions

**Conceptualization:** Marcin Dornowski.

**Data curation:** Dominika Wilczyńska, Tomasz Szot, Robert Urbański.

**Formal analysis:** Dominika Wilczyńska, Milena Lachowicz, Andriy Maznychenko, Andrzej Szwarc.

**Funding acquisition:** Piotr Sawicki.

**Investigation:** Marcin Dornowski, Dominika Wilczyńska, Milena Lachowicz, Inna Sokolowska, Kacper Gotner, Dominik Duda.

**Methodology:** Marcin Dornowski, Dominika Wilczyńska.

**Project administration:** Andrzej Szwarc, Kacper Gotner.

**Resources:** Marcin Dornowski.

**Supervision:** Marcin Dornowski, Dominika Wilczyńska.

**Software:** Tomasz Szot, Robert Urbański.

**Visualization:** Tomasz Szot.

**Writing – original draft:** Marcin Dornowski.

**Writing – review & editing:** Marcin Dornowski, Dominik Duda, Jakub Hinca.

## References

[1] Chacko E Ling B Avny N. Mindfulness-based cognitive therapy for stress reduction in family carers of people living with dementia: a systematic review. Int J Environ Res Public Health. 2022;19:614. 35010874 10.3390/ijerph19010614 PMC8744610

[2] Hafen PS Preece CN Sorensen JR. Repeated exposure to heat stress induces mitochondrial adaptation in human skeletal muscle. J Appl Physiol (1985). 2018;125:1447–55. 30024339 10.1152/japplphysiol.00383.2018

[3] Mikicin M Mróz A Karczewska-Lindinger M. Effect of the neurofeedback-EEG training during physical exercise on the range of mental work performance and individual physiological parameters in swimmers. Appl Psychophysiol Biofeedback. 2020;45:49–55. 32232604 10.1007/s10484-020-09456-1 PMC7250807

[4] Dupee M Werthner P. Managing the stress response: the use of biofeedback and neurofeedback with olympic athletes. Biofeedback. 2011;39:92–4.

[5] Conde E Filgueiras A Lacerda A. The Effects of EEG Neurofeedback Training on the Behavioral Complaints of Soccer Athletes – A Case Study: In: Proceedings of the 3rd International Congress on Sport Sciences Research and Technology Support. SCITEPRESS – Science and and Technology Publications. 2015:132–138.

[6] Romagnoli M Alis R Aloe R. Influence of training and a maximal exercise test in analytical variability of muscular, hepatic, and cardiovascular biochemical variables. Scand J Clin Lab Invest. 2014;74:192–8. 24484196 10.3109/00365513.2013.873948

[7] De La Torre G. Cognitive neuroscience in space. Life. 2014;4:281–94. 25370373 10.3390/life4030281 PMC4206847

[8] Reiter K Andersen SB Carlsson J. Neurofeedback treatment and posttraumatic stress disorder: effectiveness of neurofeedback on posttraumatic stress disorder and the optimal choice of protocol. J Nerv Ment Dis. 2016;204:69–77. 26825263 10.1097/NMD.0000000000000418

[9] Blaskovits F Tyerman J Luctkar-Flude M. Effectiveness of neurofeedback therapy for anxiety and stress in adults living with a chronic illness: a systematic review protocol. JBI Database System Rev Implement Rep. 2017;15:1765–9. 10.11124/JBISRIR-2016-003118 28708739

[10] Pinheiro J Simões De Almeida R Marques A. Emotional self-regulation, virtual reality and neurofeedback. Comput Hum Behav Rep. 2021;4:100101.

[11] Sugiono S Prasetya RP Fanani AA. Predicting the mental stress level of drivers in a braking car process using artificial intelligence. Acta Neuropsychologica. 2022;20:1–15.

[12] Smith KJ Gavey S RIddell NE. The association between loneliness, social isolation and inflammation: a systematic review and meta-analysis. Neurosci Biobehav Rev. 2020;112:519–41. 32092313 10.1016/j.neubiorev.2020.02.002

[13] Escribano D Gutiérrez AM Tecles F. Changes in saliva biomarkers of stress and immunity in domestic pigs exposed to a psychosocial stressor. Res Vet Sci. 2015;102:38–44. 26412517 10.1016/j.rvsc.2015.07.013

[14] Suarez EC Sundy JS. The cortisol: C-reactive protein ratio and negative affect reactivity in depressed adults. Health Psychol. 2017;36:852–62. 28650200 10.1037/hea0000517 PMC6029876

[15] Sproston NR Ashworth JJ. Role of C-reactive protein at sites of inflammation and infection. Front Immunol. 2018;9:754. 29706967 10.3389/fimmu.2018.00754 PMC5908901

[16] Sharpley CF Bitsika V McMillan ME. The association between cortisol:C-reactive protein ratio and depressive fatigue is a function of CRP rather than cortisol. Neuropsychiatr Dis Treat. 2019;15:2467–75. 31695383 10.2147/NDT.S213839 PMC6717724

[17] Podstawski R Borysławski K Pomianowski A. Endocrine effects of repeated hot thermal stress and cold water immersion in young adult men. Am J Mens Health. 2021;15:155798832110083. 10.1177/15579883211008339 PMC8047510 33845653

[18] Wijsman J Grundlehner B Hao Liu. Towards mental stress detection using wearable physiological sensors. In: 2011 Annual International Conference of the IEEE Engineering in Medicine and Biology Society. IEEE. 2011:1798–1801. 10.1109/IEMBS.2011.6090512 22254677

[19] Shivpuri S Gallo LC Crouse JR. The association between chronic stress type and C-reactive protein in the multi-ethnic study of atherosclerosis: does gender make a difference? J Behav Med. 2012;35:74–85. 21503709 10.1007/s10865-011-9345-5 PMC3268954

[20] Kennedy E Niedzwiedz CL. The association of anxiety and stress-related disorders with C-reactive protein (CRP) within UK Biobank. Brain Behav Immun Health. 2022;19:100410. 35028602 10.1016/j.bbih.2021.100410 PMC8741412

[21] Radua J Stoica T Scheinost D. Neural correlates of success and failure signals during neurofeedback learning. Neuroscience. 2018;378:11–21. 27063101 10.1016/j.neuroscience.2016.04.003 PMC5537049

[22] Glombiewski JA Bernardy K Häuser W. Efficacy of EMG- and EEG-biofeedback in fibromyalgia syndrome: a meta-analysis and a systematic review of randomized controlled trials. Evid-Based Complement Alternat Med. 2013;2013:1–11. 10.1155/2013/962741 PMC3776543 24082911

[23] Tan G Thornby J Hammond DC. Meta-analysis of EEG biofeedback in treating epilepsy. Clin EEG Neurosci. 2009;40:173–9. 19715180 10.1177/155005940904000310

[24] Arns M De Ridder S Strehl U. Efficacy of neurofeedback treatment in ADHD: the effects on inattention, impulsivity and hyperactivity: a meta-analysis. Clin EEG Neurosci. 2009;40:180–9. 19715181 10.1177/155005940904000311

[25] Stanton R Ada L Dean CM. Biofeedback improves activities of the lower limb after stroke: a systematic review. J Physiother. 2011;57:145–55. 21843829 10.1016/S1836-9553(11)70035-2

[26] Bazanova OM Auer T Sapina EA. On the efficiency of individualized theta/beta ratio neurofeedback combined with forehead EMG training in ADHD children. Front Hum Neurosci. 2018;12:3. 29403368 10.3389/fnhum.2018.00003 PMC5785729

[27] Kiss B Balogh L. Original Article A study of key cognitive skills in handball using the Vienna test system. Article in J Phys Educ Sport. 2019;19:733–41.

[28] Marzbani H Marateb H Mansourian M. Methodological note: neurofeedback: a comprehensive review on system design, methodology and clinical applications. Basic Clin Neurosci J. 2016;7:143–58. 10.15412/J.BCN.03070208 PMC4892319 27303609

[29] Ros T Moseley MJ Bloom PA. Optimizing microsurgical skills with EEG neurofeedback. BMC Neurosci. 2009;10:87. 19630948 10.1186/1471-2202-10-87 PMC2723116

[30] Norouzi E Hossieni F Solymani M. Effects of neurofeedback training on performing bimanual coordination in-phase and anti-phase patterns in children with ADHD. Appl Psychophysiol Biofeedback. 2018;43:283–92. 30073605 10.1007/s10484-018-9408-2

[31] Xiang MQ Hou XH Liao BG. The effect of neurofeedback training for sport performance in athletes: a meta-analysis. Psychol Sport Exerc. 2018;36:114–22.

